# Endovascular management of spontaneous axillary artery aneurysm: a case report and review of the literature

**DOI:** 10.1186/1752-1947-7-140

**Published:** 2013-05-28

**Authors:** Changsheng He, Xingjiang Wu, Jianmin Cao, Xinxin Fan, Kai Liu, Baochen Liu

**Affiliations:** 1 Institute of General Surgery, Nanjing Jinling Hospital, 305 East Zhongshan Road, Nanjing 210002, China; 2 Department of Radiology, Nanjing Jinling Hospital, 305 East Zhongshan Road, Nanjing 210002, China; 3 Clinical School of Nanjing University, 305 East Zhongshan Road, Nanjing 210002, China

**Keywords:** Axillary artery aneurysm, Endovascular management

## Abstract

**Introduction:**

Spontaneous atraumatic true axillary artery aneurysm is a relatively unusual disorder. Although most cases are asymptomatic, complications of axillary artery aneurysms may result in acute vascular insufficiency and neurological deficits. Prompt treatment, therefore, should be employed in the management of this condition. To date, the standard treatment for peripheral aneurysms is still surgical resection with end-to-end anastomosis. However, aneurysmectomy and interposition grafting with autologous or artificial vessels are more invasive and time-consuming. The ideal treatment for axillary artery aneurysm should be relatively noninvasive, safe and free of significant complications, cost-effective, cosmetically acceptable, and incur less absence from usual daily activities. Endovascular stent grafts have also been successfully used to treat these aneurysms. Management of select aneurysms using stent grafts has become more prevalent with the developing endoluminal technology.

**Case presentation:**

We report a case of a spontaneous atraumatic axillary artery aneurysm where the patient was a 48-year-old ethnic Han Chinese woman with a gradually enlarging left axillary pulsatile mass. She was treated with endovascular stent grafts. The postoperative course of the patient was uneventful during the six-month follow-up.

**Conclusions:**

We show that there are significant early advantages with the endovascular management technique versus the conventional operation in the management of axillary artery aneurysm.

## Introduction

Arterial aneurysms can occur in any vascular territory. Aneurysms of the upper extremities are rare in relation to other peripheral aneurysms. In spite of their rarity, upper extremity aneurysms can result in profound disability secondary to inadequate diagnosis and management. The uncommon nature of aneurysms in this location lends the upper extremity symptoms to be ascribed to an alternative etiology. However, with the advent of more sophisticated imaging modalities, these aneurysms are now less likely to be missed. Spontaneous atraumatic true axillary artery aneurysm is a relatively unusual disorder. There have been a few reports in the literature describing atraumatic atherosclerotic aneurysms of the axillary artery. In this paper, we report a case of a spontaneous atraumatic axillary artery aneurysm that was treated with endovascular stent grafts in a patient with no history of prior trauma.

## Case presentation

**Figure 1:**
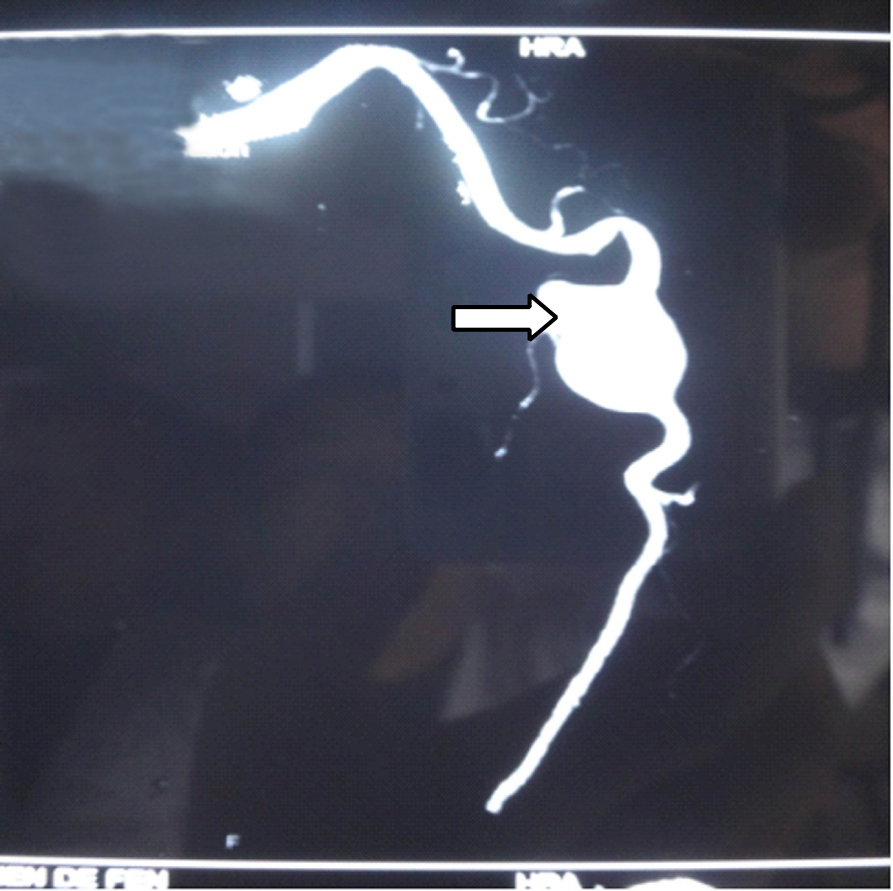
**Computed tomography (CT) angiography revealed a 3**×**5cm size true aneurysm of the left axillary artery (arrowed).**

**Figure 2:**
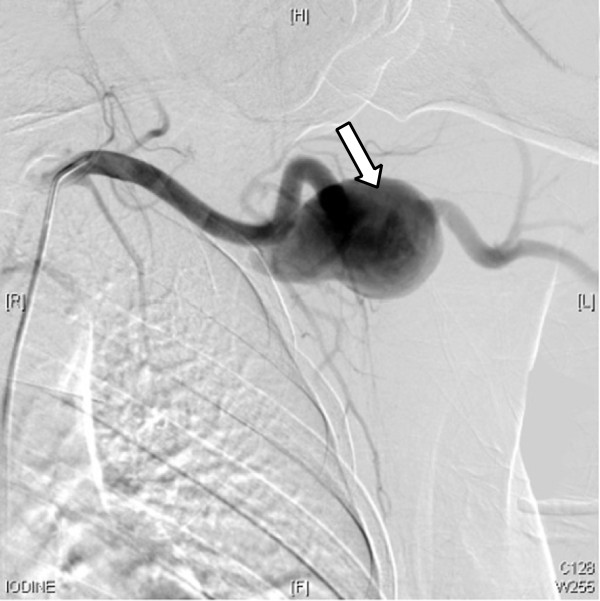
Digital subtracted angiogram showed a 3×5cm fusiform aneurysm arising from the proximal left axillary artery (arrowed)

**Figure 3:**
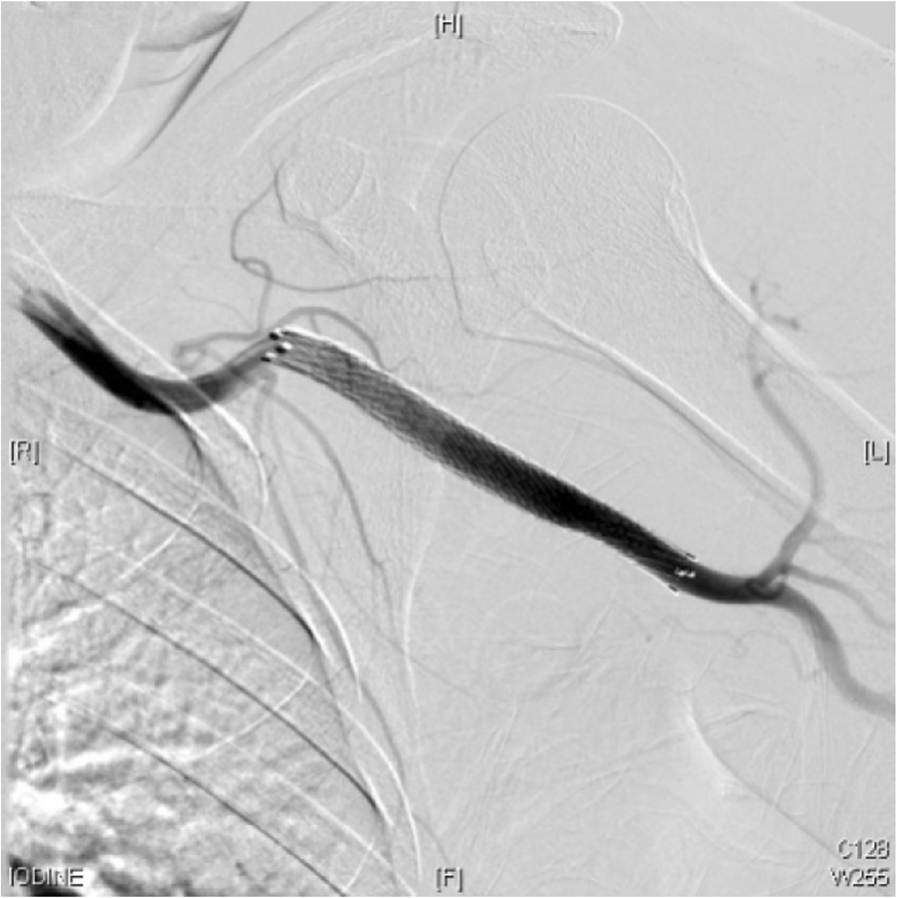
Completion angiography of the left axillary artery showed good flow through the stent graft and complete exclusion of the aneurysm.

**Figure 4:**
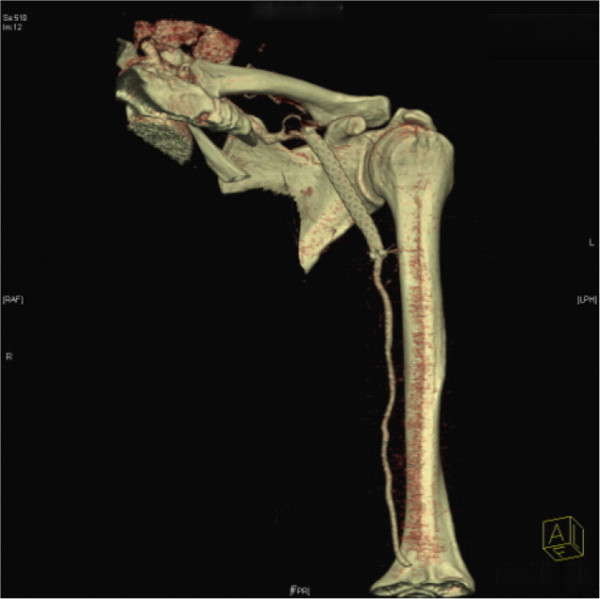
Computed tomography (CT) angiography showed complete exclusion of the aneurysm without any endoleaks.

A 48-year-old ethnic Han Chinese woman was admitted to our hospital with a gradually enlarging left axillary pulsatile mass. She complained of left upper extremity weakness, and pain. She had a past medical history significant for hypertension. She denied a history of previous upper extremity or chest trauma. She had noticed the mass two months before, and it had been gradually enlarging since then. In our physical examination, we found an about 3×5cm solid, palpable pulsating mass in the left axillary fossa. Brachial blood pressures were 100/60mmHg on her left side and 145/90mmHg on her right side. Upper extremity radial pulse in her left side was weak in comparison with the opposite side. On auscultation, a systolic murmur, which was spreading to the left axillary region, was heard on the mass. A neurological examination of her left arm was normal. There were no signs of vasculitis or connective tissue diseases associated with arterial involvement such as hyperelastic skin, hypermobile joints, or marfanoid habitus. Her laboratory test results, including erythrocyte sedimentation rate, C-reactive protein, complete blood count, serological test for syphilis, rheumatoid factor, antinuclear antibody, antithrombin III, protein C, and protein S, were normal. A computed tomography (CT) angiography revealed a 30×51mm size true aneurysm of the left axillary artery (Figure 1). A color Doppler ultrasound suggested the presence of an axillary artery aneurysm. Digital subtraction angiography confirmed a 3×5cm fusiform aneurysm at the proximal part of her left axillary artery (Figure 2). The aneurysm extended to her left axillary artery distally. Thoracic outlet syndrome was excluded by means of careful clinical, radiological, and electrophysiological examinations. We planned to adopt a less invasive approach using a stent graft of the artery. Under local anesthesia, percutaneous access was obtained through the right groin and a 7-French sheath was introduced through the side. A 5-French pigtail catheter was advanced into the ascending thoracic aorta. Angiography confirmed the patency of the left axillary artery. The size and position of the covered stent graft were assessed on the angiogram. The covered stent graft with a diameter of 8mm and a length of 80mm (Cook Medical Inc., Bloomington, IN, USA) was reloaded in a reverse manner using umbilical tape. The stent graft was deployed from the distal to the proximal site of the left axillary artery excluding the origin of the subclavian artery. Completion angiography of the left axillary artery showed good flow through the stent graft and complete exclusion of the aneurysm (Figure 3). There were no adverse peri-operative events, including ischemic symptoms of the left arm. CT angiography showed complete exclusion of the aneurysm without any endoleaks (Figure 4). The postoperative course of the patient was uneventful and the patient was discharged on the fifth postoperative day. The patient was followed up for six months. During follow-up, she was symptom-free and no further studies were needed.

## Discussion

Axillary artery aneurysm is the rarest peripheral artery aneurysm. Most of them are asymptomatic [1]. The natural history of axillary artery aneurysms is not well known due to the limited number of cases. However, with the advent of more sophisticated imaging modalities, these aneurysms are now less likely to be missed. Aneurysms of the axillary artery are generally secondary to blunt or penetrating trauma, causing arterial degeneration. This includes the use of axillary crutches or high-stress overhead arm motions during athletic activities [2]. Infectious and degenerative causes are unusual, but have been reported [3,4]. Atherosclerosis, collagen vascular disease, advanced age, mycotic aneurysm and thoracic outlet syndrome have also been described as etiologies [5,6]. The recognition and treatment of these aneurysms are important because they tend to increase in size with an increased risk of rupture, thrombosis, embolization and compression of adjacent structures. No reports have documented any correlation between the absolute size of aneurysms and the potential risk for rupture, but if left untreated, rupture with life-threatening blood loss, thromboembolism leading to ischemia and limb loss, or irreversible neurological damage may occur. For this reason, prompt diagnosis and intervention is mandatory for axillary aneurysms even when they are asymptomatic. To date, the standard treatment for peripheral aneurysms is still surgical resection with end-to-end anastomosis, if necessary with autologous or artificial patch repair [1,6]. Complications, such as thrombosis, embolism, rupture and rapid growth with increasing symptoms, are absolute indications of surgery. However, aneurysmectomy and interposition grafting with autologous or artificial vessels are more invasive. In addition, brachial or axillary veins may tend to develop aneurysms, and saphenous vein size matching may be difficult [1,5,6]. The ideal treatment for axillary artery aneurysm should be relatively noninvasive, safe and free of significant complications, cost-effective, cosmetically acceptable, and incur less absence from usual daily activities. Endovascular stent grafts have also been successfully used to treat these aneurysms [7]. They are a good alternative when the quality of the saphenous vein is inadequate or lacking. Most can be treated effectively with surgical excision and vascular grafting. The management of select aneurysms using stent grafts has become more prevalent with the developing endoluminal technology [8-11]. It can be an effective and less invasive alternative to the standard surgical reconstruction, but only a few cases have been described in the literature with respect to axillary artery aneurysms. There has been only one case report that is similar to ours [7]. In our case, an 8cm stent was placed successfully. There were no adverse peri-operative events including any ischemic symptoms of the left arm. A CT angiogram showed complete exclusion of the aneurysm without any endoleaks. The postoperative course of the patient was uneventful and the patient was discharged on the fifth postoperative day. On follow-up, the patient was symptom-free and no further studies were needed. We have shown that there are significant early advantages with this technique versus the conventional operation in the management of axillary artery aneurysm. We think that endovascular management of spontaneous axillary artery aneurysm is better than conventional surgical reconstruction. Although stenting across a mobile joint, such as the shoulder, can lead to problems like deformation and fractures of the stent, with the advent of stent grafts with increased flexibility, less compressibility and deformability, the role of endovascular treatment of peripheral aneurysms is promising. We recommend using this technique to deal with axillary artery aneurysm rather than the conventional operation.

## Conclusions

Axillary artery aneurysm is a rare disorder. Open surgical therapy has been the standard of care with excellent results, but endovascular management is an evolving option. Endovascular therapy is a minimally invasive technique. In addition, it is an effective and safe therapeutic approach when performed by an experienced vascular surgeon. It also has the advantage of shortening the hospital stay. In conclusion, with its higher technical success rate and lower mortality and morbidity rates, it is superior to open surgical therapy. Further clinical studies, however, are needed. Long-term studies are needed to determine the ultimate fate of the axillary artery aneurysm via endovascular management.

## Consent

Written informed consent was obtained from the patient for publication of this manuscript and any accompanying images. A copy of the written consent is available for review by the Editor-in-Chief of this journal.

## Competing interests

The authors declare they have no competing interests.

## Authors’ contributions

LK, FX and LB analyzed and interpreted the data from our patient regarding the blood tests and the disease. HC, WX and CJ made the diagnosis of axillary artery aneurysm and established the treatment. HC was a major contributor in writing the manuscript. All authors read and approved the final manuscript.
